# **Retraction: Regional effects of the renewable energy components on CO**_**2**_ **emissions of Asia-Pacific countries**

**DOI:** 10.1371/journal.pone.0323144

**Published:** 2025-05-07

**Authors:** The PLOS One Editors

The *PLOS One* Editors retract this article [1] due to concerns about potential manipulation of the publication process. These concerns call into question the validity and provenance of the reported results. We regret that the issues were not identified prior to the article’s publication.

All authors either did not respond directly or could not be reached.
